# Assessing the Efficacy of Ventilation of Anesthetized Neonatal Calves Using a Laryngeal Mask Airway or Mask Resuscitator

**DOI:** 10.3389/fvets.2018.00292

**Published:** 2018-11-21

**Authors:** Laura Armstrong, Nigel Caulkett, Søren Boysen, Jennifer M. Pearson, Cameron G. Knight, M. Claire Windeyer

**Affiliations:** ^1^Department of Production Animal Health, University of Calgary Faculty of Veterinary Medicine, Calgary, AB, Canada; ^2^Department of Veterinary Clinical and Diagnostic Sciences, University of Calgary Faculty of Veterinary Medicine, Calgary, AB, Canada

**Keywords:** neonate, calf, ventilation, resuscitation, airway

## Abstract

Calves that have undergone a dystocia are often hypoxic and acidemic, which can result in reduced vigor and subsequent mortality. Methods of field resuscitation of apneic newborn calves are often ineffective and therefore underutilized. This proof-of-concept study aimed to determine the efficacy of the laryngeal mask airway (LMA) as well as the current industry standard method of ventilation, the McCulloch Calf Aspirator/ Resuscitator (MMR) for positive pressure ventilation of neonatal calves. Five LMA models of various sizes were first tested in cadaver heads to assess anatomical fit. Three LMA models in two sizes each were then tested in two anesthetized calves to determine the model best suited to ventilate calves. Next, the selected LMA and the MMR were both assessed for efficacy of ventilation. Six anesthetized calves had hypoventilation induced by administering alfaxalone intravenously. Calves were ventilated for 3 min with the LMA, allowed a brief washout period, then given a second administration of alfaxalone prior to ventilation with the MMR. Serial arterial blood gas analyses were performed prior to ventilation (baseline), at 1, 2, and 3 min during ventilation, and 1 min after ventilation had ceased. Success of ventilation was assessed by monitoring partial pressure of oxygen (PaO_2_), partial pressure of carbon dioxide (PaCO_2_), bicarbonate (${\text{HCO}}_{3}^{-}$), pH, L-lactate, and hemoglobin saturation (SaO_2_) in arterial blood. A one-way ANOVA for repeated measures with Bonferroni correction was used to assess the efficacy of ventilation of each device compared to baseline. For the LMA, PaO_2_, SaO_2_, and pH were significantly higher than baseline throughout ventilation and PaCO_2_ was significantly lower than baseline at 1 min of ventilation. For the MMR, PaO_2_ and SaO_2_ were significantly higher and PaCO_2_ and ${\text{HCO}}_{3}^{-}$ were significantly lower than baseline for 1 to 2 min of ventilation. This proof-of-concept study showed the LMA is an effective means of ventilating neonatal calves, as was the MMR.

## Introduction

Parturition is a time of substantial risk for the cow and calf, with two of the major problems encountered being dystocia and perinatal mortality (1). Dystocia is defined as a difficult or delayed birth, and perinatal mortality as calf death during or within 48 h of parturition, following a gestation period of a minimum of 260 days (2). These two problems are interconnected, as calves that have undergone a dystocia are often born weak and are unable to effectively aerate their lungs (3). The most critical aspect of the transition to extrauterine life is probably the commencement of respiration to allow oxygenation of the blood (4). After the rupture of the umbilical cord, hypoxia, hypercapnia, and acidemia stimulate gasping reflexes and spontaneous breathing should begin (4, 5). If self-ventilation is not quickly achieved by the calf, the consequence is typically death (6), because effective intervention is rarely achieved in field situations.

Neonatal mortality due to dystocia is an important economic and social concern. Birth-related problems accounted for the greatest proportion (25.6%) of deaths in beef calves within the first 3 weeks of life (7). In dairy calves, 5.3% of preweaning mortality is attributed to calving problems (8). The estimated economic impact from the loss of heifer calves from dystocia in US dairies alone is $125 million annually (9). Thus, post-dystocia resuscitation of the calf is an important measure to decrease calf losses in both the beef and dairy industries (5).

In neonatal calves, resuscitation focuses on ventilation and correction of acid-base imbalances (5). Stimulation of spontaneous respiration in calves has been attempted by rubbing the calf with towels or blankets, initiating a gasping reflex by placing a finger in the nose, or pouring cold water over the calf's head (5, 10). When attempts to promote spontaneous respiration fail, mechanical ventilation may be the last resort to save the calf. Positive pressure ventilation is the only means of overcoming the surface tension of alveoli and elastic recoil in the lung tissue to aerate lungs (11).

Methods of positive pressure ventilation that are currently employed in the field include mouth-to-nose or mouth-to-mouth resuscitation, nasal tubes, and facial masks. The McCulloch Calf Aspirator/ Resuscitator (MMR; McCulloch Medical, MAI Animal Health, Wisconsin, United States) is a mask resuscitator considered to be the current standard of care for positive pressure ventilation of neonatal calves in the field (2). This facial mask works by pushing air into the oro-nasal cavity through a bi-directional valve that allows the animal to exhale without removing the mask from their face; however, it tends to push air into the esophagus and does not protect the airway from aspiration. Unfortunately, none of the currently available methods of ventilation are adept at providing an adequate air-tight seal and some of the options put the caregiver at risk of zoonotic diseases. There is also no effective means to prevent air from entering the esophagus and distending the abomasum (5). The use of a cuffed endotracheal tube has been described as an effective means of positive pressure ventilation in newborn calves (5, 11). However, this method is technically challenging to perform, especially in a field setting, and is unlikely to be adopted by producers as most calves requiring resuscitation are not attended by a veterinarian (2).

Laryngeal mask airways (LMAs) were designed for humans as an alternative to endotracheal intubation when visualization of the larynx is difficult or not achievable (12). The LMA can be considered a hybrid between a face mask and an endotracheal tube, where a silicone mask with an inflatable cuff overlies the laryngeal entrance, creating a seal to allow for ventilation of the lungs without air entering the esophagus. One study in human patients showed that unskilled personnel who were trained in inserting endotracheal (ET) tubes as well as an LMA had a 100% success rate at first insertion with the LMA, compared with a 0% success rate for first attempt with an ET tube (13). The effectiveness of LMAs has been demonstrated in veterinary medicine for the ventilation of cats, rabbits, pigs, and dogs under anesthesia (14–17).

Timely and effective resuscitation is imperative for revival of calves that are apneic due to a dystocia. The LMA may prove to be a valuable tool for neonatal calf resuscitation in the field. Because positive pressure ventilation is key for resuscitation, the objective of this study was to assess the efficacy of the LMA, and the current standard for field resuscitation, the MMR, for neonatal calf ventilation. It was hypothesized that the LMA but not the MMR would be efficacious for ventilating neonatal calves.

## Materials and methods

This study was conducted in accordance with the guidelines of the Canadian Council on Animal Care and was approved by the Veterinary Sciences Animal Care Committee of the University of Calgary (protocol #AC17-0173). This study used nine neonatal Holstein bull calves between the ages of 2 to 11 days old, ranging from 37 kg to 54 kg, sourced from 4 Alberta dairy herds.

### Stage one: cadaver study

Five models of LMA and a Bag-Valve-Mask (BVM) resuscitator (Adult size 1.5L bag) from Intersurgical Ltd (Crane House, Wokingham, Berkshire, United Kingdom) were provided for trial placement in cadaver calf heads prior to initiating the live animal portion of the study. The different LMA models (iGel® sizes 4 and 5, LMA Classic Excel® size 3, LMA Unique® size 3, Solus® sizes 4 and 5, and Solus Flexible® sizes 4 and 5) were placed in the cadavers, using a 30cm, plastic-coated, bendable metal stylet if necessary. The cuff was inflated if applicable to that model and the distal end of tube was attached to the BVM. Ventilation with the BVM and LMA was evaluated by placing a medium-sized latex glove around the exposed trachea of the cadaver and attempting to inflate the glove. Radiographs were taken in dorsal-ventral and lateral views to evaluate the placement of the LMA. Three models of LMA were selected for use in the live animal portion of the study based on radiographic positioning, ease of placement in the cadavers, and ability to form a seal around the larynx and inflate the latex glove.

### Stage two: selection of LMA model

Based on Stage One, 3 different models of LMA (iGel®, Solus Flexible®, and Solus®) in 2 sizes each (size 4 and 5) were evaluated in Stage Two. Calves were not fed the morning of the experiment. Two, 4- to 5-day old, healthy Holstein bull calves (Calves 1 and 2) were anesthetized with 0.2 mg/kg xylazine (20 mg/ml; Rompun®, Bayer Animal Health, Mississauga Ontario, Canada) and 3 mg/kg alfaxalone (10 mg/ml; Alfaxan®, Central Sales Ltd., Brampton Ontario, Canada) administered intramuscularly. Once sedated, each calf was placed in sternal recumbency with the head and neck supported by one person. Prior to insertion of the LMA, the cuff was deflated and lubricating gel was placed on the back of the cuff. A second person immobilized the tongue with one hand and used the other hand to introduce the LMA to the oral cavity and move it along the dorsal aspect of the tongue. Once over the root of the tongue and a point of resistance was met, the LMA was deemed to be in place and the cuff was inflated if applicable to that model. Once the LMA was in position, a pulse oximeter was placed on the tongue, and a capnograph (CARDELL Veterinary Monitor, Model 9405, Dispomed ltd, Quebec, Canada) and BVM were attached to the end of the tube. Using light pressure on the BVM so as to not over-inflate the lungs, positive pressure ventilation was initiated at a rate of 20 to 30 breaths per minute. A single operator (LA) was used throughout and efforts were made to maintain a consistent level of pressure for each breath. Each model and size of LMA were evaluated based on ease of placement (including the need of a stylet), the seal formed around the larynx, excursion of the chest wall, presence of borborygmi (auscultated with a stethoscope at the level of the abomasum that would indicate aerophagia), maintenance of SpO_2_ >90%, and the ability to lower end-tidal CO_2_ (EtCO_2_) to below 40 mmHg within 5 min of the start of ventilation.

### Stage three: evaluation of the LMA and MMR

The selected model of LMA from Stage Two was assessed along with the current industry standard method for positive pressure ventilation, the MMR. Seven, 2- to 11-day old, healthy neonatal Holstein bull calves (Calves 3 through 9) were anesthetized with the same protocol as Stage Two of the study. Once sedated, a 1-inch 18 g intravenous catheter was placed in the cephalic vein for drug administration. For serial arterial blood sampling, attempts to pass a 1-inch 22 g catheter into the auricular artery were first made. If placement of the auricular arterial catheter was not achieved within 10 min, a femoral arterial catheter was placed via a mini cutdown. Therefore, all calves either had an auricular arterial (Calves 4, 8, 9) or femoral arterial (Calves 3, 5, 6, 7) catheter for the duration of the study (i.e., sampling site did not vary within a particular calf). Once both intravenous and arterial catheters had been placed, hypoventilation was induced by administering 2 mg/kg alfaxalone (10 mg/ml) intravenously.

Initially, the order of use of the LMA or MMR were randomized. A coin was flipped, and Calf 3 (the first calf used for Stage Three) was designated to be ventilated first with the LMA and then the MMR. The intent was to then ventilate each calf starting with alternating devices (i.e., Calf 4 with the MMR first, Calf 5 with the LMA first, and so on). This pattern was continued until Calf 6, who was ventilated for 3 min with the MMR before becoming noticeably bloated and subsequently regurgitating gastric content during the placement of the LMA. Subsequent calves were ventilated with LMA prior to the MMR.

The LMA was placed using the same technique from Stage Two. Calves were monitored with a pulse oximeter placed on the tongue and capnograph placed between the end of the LMA tube and the BVM. A baseline arterial blood sample was collected then calves were immediately ventilated at a rate of 20 to 30 breaths per minute.

Serial blood gas analyses were performed prior to ventilation (baseline), at 1, 2, and 3 min during ventilation, and 1 min after ventilation had ceased. Arterial blood was collected from either the auricular or femoral catheter into a 3 ml heparinized syringe. Samples were placed in ice water and analyzed within 10 min of collection by a handheld blood gas analyzer (Abaxis VetScan iSTAT analyzer, Abaxis Associated Veterinary Purchasing, Langley, British Columbia, Canada) using Abaxis CG4+ cartridges. Arterial partial pressure in oxygen (PaO_2_), arterial partial pressure in carbon dioxide (PaCO_2_), bicarbonate (${\text{HCO}}_{3}^{-}$), blood pH, L-lactate, and arterial hemoglobin saturation (SaO_2_) were measured. The values of PaO_2_, PaCO_2_, and pH were corrected for rectal temperature.

Once ventilation with the LMA had ceased and all the blood gas samples were collected, the calves were given a wash-out period with supplemental flow-by nasal oxygen at a flow rate of 3 L/min, using a Sequel Eclipse Oxygen Concentrator (CPAP Machines, Ontario, Canada). End-tidal CO_2_ (etCO_2_) was monitored via capnography and peripheral capillary oxygen saturation (SpO2) via pulse oximetry during a washout period of at least 1 min to ensure etCO_2_ was below 40 mmHg and Sp0_2_ was above 90%. Another dose of intravenous alfaxalone at 2 mg/kg (10 mg/ml) re-induced hypoventilation and the protocol was repeated using the MMR. Due to the presence of a mask over the rostral aspect calf's head, a pulse-oximeter, and capnograph were not used to evaluate the efficacy of the MMR. Serial blood gas analyses were performed as previously described.

### Pathologic examination

In both Stages Two and Three of the study, calves were kept in a deep plane of anesthesia and after all the required data had been collected, were euthanized using the A&S Cash Special 0.22R Captive Bolt (SKU: 4100R; McCordik, Cambridge, Ontario, Canada). All calves were submitted to the University of Calgary's Diagnostic Services Unit for same-day post mortem examination by a single pathologist (CK). In Calves 1 and 2 from Stage Two, the anatomic placement of the LMA was verified post-mortem. For all calves, the oropharynx, larynx, laryngopharynx and proximal esophagus were examined for evidence of trauma caused by placement of the LMA. Tissues were collected for histologic examination and transverse sections through the laryngopharyngeal floor examined microscopically.

### Statistical analysis

Statistical analyses were performed using GraphPad Prism 7® software. Parameters were assessed for normality using the Kolmogorov-Smirnov test. A one-way ANOVA for repeated measures with Bonferroni correction was conducted to compare the mean differences from baseline for blood parameters at 1, 2, and 3 min of ventilation, and 1 min after ventilation for both the LMA and the MMR. A *p*-value < 0.05 was considered significant. *Post-hoc* power analysis was performed using mean PaCO_2_ and standard deviation values at 3 min of ventilation, an alpha of 0.05, and power of 80%.

## Results

### Stage one

**Figure 1:**
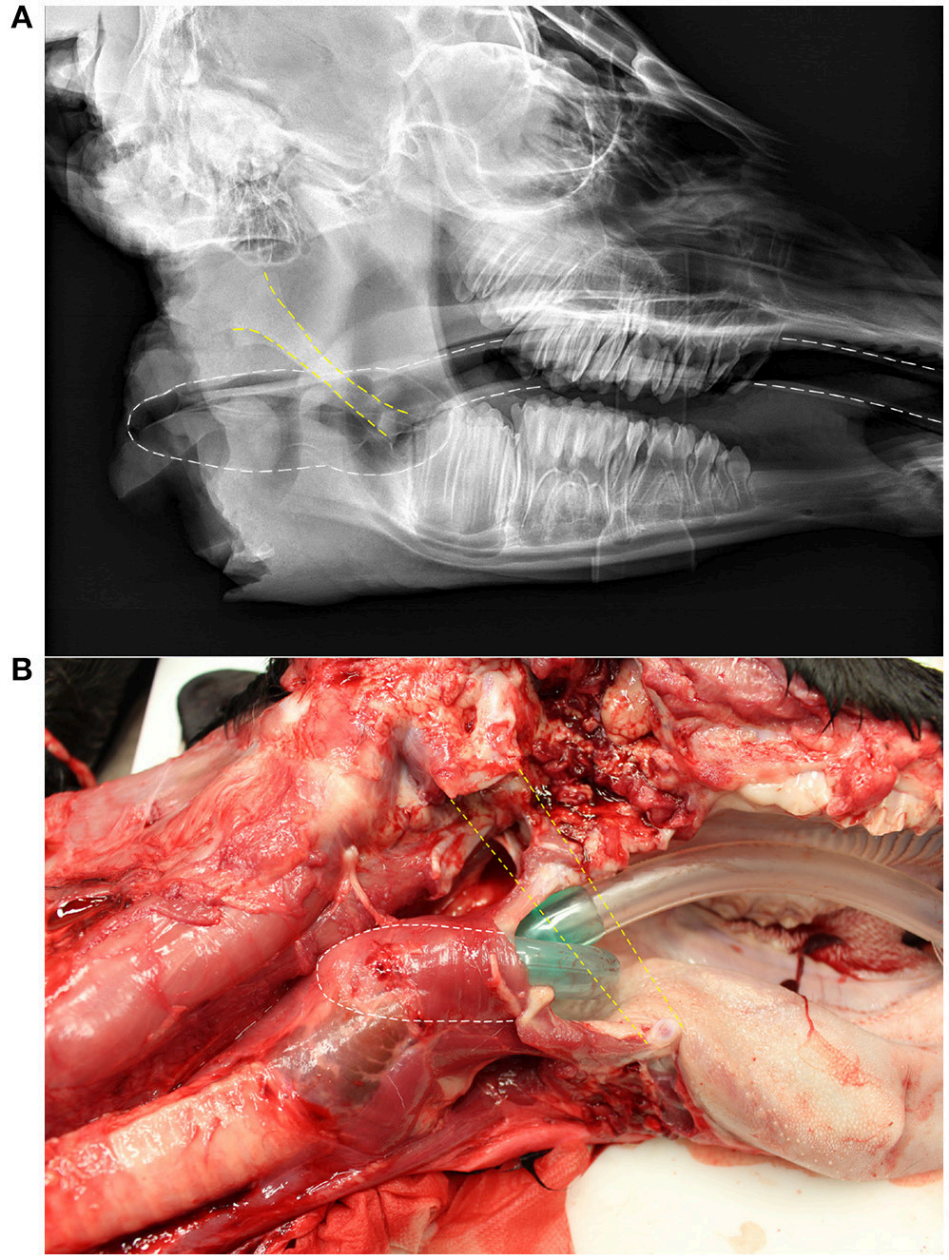
Lateral radiograph **(A)** and a dissected calf **(B)** showing the position of a laryngeal mask airway (LMA), the Solus® size 4. Yellow dashed lines indicate the position of the stylohyoid bone, which has been removed in **(B)**. White dashed lines indicate the position of the LMA.

The three models of LMA chosen for the live animal portion of the study (Stage Two and Three) were sizes 4 and 5 each of the Solus®, iGel®, and Solus Flexible®. All of these models were easily placed, though the Solus Flexible required a stylet for placement. These models were able to form a seal around the larynx of a cadaver and inflate a latex glove placed around the trachea. The position of the LMA around the larynx was confirmed by radiographic imaging (Figure 1A).

### Stage two

The model of LMA chosen from Stage Two was the Solus® in both sizes 4 and 5 (Figure 1B). This model was superior to the iGel® and Solus Flexible® for ease of placement, seal formed around the larynx, excursion of the chest wall, no borborygmi ausculted that corresponded temporally with inhalation, maintaining SpO_2_ > 90%, and the ability to lower EtCO_2_ to <40 mmHg within 5 min of ventilating. The iGel® was relatively easy to place in the anesthetized calves. However, the seal formed around the larynx was deemed inadequate because air was felt and heard leaving the oral cavity when positive pressure ventilation was initiated with the BVM. The Solus Flexible® had the longest tube, which was desirable for placement in the long oral cavity of the calves. However, the requirement to use a stylet for placement was deemed to make it unfit for the ultimate goal of calf resuscitation in the field.

### Stage three

Alfaxalone successfully induced severe hypoventilation each time it was administered so that calves were apneic and completely controlled by positive pressure ventilation during both LMA and MMR ventilation. Also, all calves re-initiated spontaneous ventilation during the washout period. Calf 6 was removed from the dataset for statistical analysis due to aspiration of gastrointestinal content while being ventilated with the MMR, confirmed by post mortem examination. Subsequent data collected was considered inaccurate for that calf and was therefore removed. *Post-hoc* power analysis, using mean PaCO_2_ values of 33.6 and 44.8 mmHg, and standard deviation of 7.0, resulted in a required sample size of 9 animals.

All parameters were normally distributed. The comparisons between mean baseline values for the arterial blood parameters and mean values at 1, 2, and 3 min of ventilation, and 1 min after ventilation with each device as assessed by one-way ANOVA with Bonferroni correction are presented in Table 1. For the LMA, PaO_2_, SaO_2_, and pH were significantly higher than baseline at 1, 2, and 3 min of ventilation, and PaCO_2_ was significantly lower than baseline at 1 min of ventilation (*p* < 0.05). For the MMR, PaO_2_ was significantly higher and PaCO_2_ was significantly lower than baseline at 1 min of ventilation, SaO_2_ was significantly higher than baseline at 1 and 2 min of ventilation, and ${\text{HCO}}_{3}^{-}$ was significantly lower than baseline at 1 and 2 min of ventilation (*p* < 0.05).

**Table 1:** Comparison between baseline mean (95% and L-lactate confidence interval) arterial partial pressure of oxygen (PaO_2_), partial pressure of carbon dioxide (PaCO_2_), hemoglobin saturation (SaO_2_), pH, bicarbonate (HCO_3_-) measured in six neonatal calves and the mean values at 1, 2, and 3 min of ventilation, and 1 min post-ventilation with either a laryngeal mask airway (LMA) or McCulloch Calf Aspirator/ Resuscitator (MMR).

	**Baseline**	**1 min of ventilation**	**2 min of ventilation**	**3 min of ventilation**	**1 min post-ventilation**
**LMA**					
PaO_2_ (mmHg)	28.5 (23.4–33.6)^a^	61.3 (44.6–8.1)^b^	70.2 (54.9–85.4)^b^	73.0 (54.7–91.4)^b^	24.8 (20.5–29.2)^a^
PaCO_2_ (mmHg)	53.8 (48.6–59.0)^a, c^	42.1 (40.7–43.5)^b^	37.9 (31.9–44.0)^a, b^	35.6 (28.4–42.7)^a, b^	48.1 (44.2–52.2)^c^
SaO_2_ (%)	45.3 (32.3–58.4)^a^	88.5 (82.14–94.9)^b^	93.5 (89.5–97.5)^b^	94.5 (91.4–97.6)^b^	39.8 (28.0–51.7)^a^
pH	7.38 (7.34–7.42)^a^	7.46 (7.41–7.51)^b, c^	7.52 (7.43–7.61)^b^	7.55 (7.44–7.65)^b^	7.44 (7.38–7.50)^a, c^
HCO_3_- (mEq/L)	31.1 (27.7–34.6)^a, b^	29.9 (27.0–32.8)^a^	30.2 (28.5–32.0)^a^	30.7 (29.2–32.2)^a, b^	32.7 (30.6–34.7)^b^
L–Lactate (mmol/L)	0.99 (0.37–1.62)^a^	1.08 (0.47–1.69)^a^	1.14 (0.48–1.79)^a^	1.14 (0.51–1.78)^a^	1.09 (0.52–1.66)^a^
**MMR**					
PaO_2_ (mmHg)	26.7 (18.8–34.6)^a, c^	50.8 (38.5–63.1)^b^	58.0 (39.7–76.3)^a, b^	56.7 (31.5–81.9)^a, b, c^	22.3 (14.5–30.2)^c^
PaCO_2_ (mmHg)	58.5 (53.3–63.7)^a^	49.2 (44.8–53.6)^b^	47.4 (42.7–52.1)^a, b^	44.8 (36.1–53.5)^a, b^	54.2 (49.7–58.7)^a, b^
SaO_2_ (%)	41.0 (22.8–59.2)^a, c^	80.7 (69.6–91.8)^b^	84.7 (73.5–95.9)^b^	78.0 (49.0–107.0)^a, b^	33.5 (15.6–51.4)^c^
pH	7.37 (7.33–7.41)^a, b^	7.42 (7.37–7.47)^a, b^	7.45 (7.39–7.51)^a^	7.46 (7.39–7.53)^a, b^	7.40 (7.35–7.44)^b^
HCO_3_- (mEq/L)	33.8 (31.2–36.5)^a^	31.3 (28.5–34.0)^b^	31.3 (28.8–33.8)^b^	31.2 (27.6–34.7)^a, b^	32.9 (30.6–35.1)^a, b^
L–Lactate (mmol/L)	1.10 (0.46–1.74)^a^	1.11 (0.55–1.68)^a^	1.15 (0.52–1.77)^a^	1.14 (0.51–1.77)^a^	1.12 (0.53–1.71)^a^

*A one-way ANOVA for repeated measures with Bonferroni correction was conducted to compare among time points for each device. Values within a row that do not share a common superscript are statistically different, using a p < 0.05 as the threshold for significance*.

Because of the issues with randomization, comparisons between the LMA and MMR could not be made due to temporal confounding whereby most calves had been under anesthesia for a longer period of time during MMR ventilation then during LMA ventilation. This was deemed likely to impact the blood gas parameters and make any comparisons invalid.

### Pathologic examination

**Figure 2:**
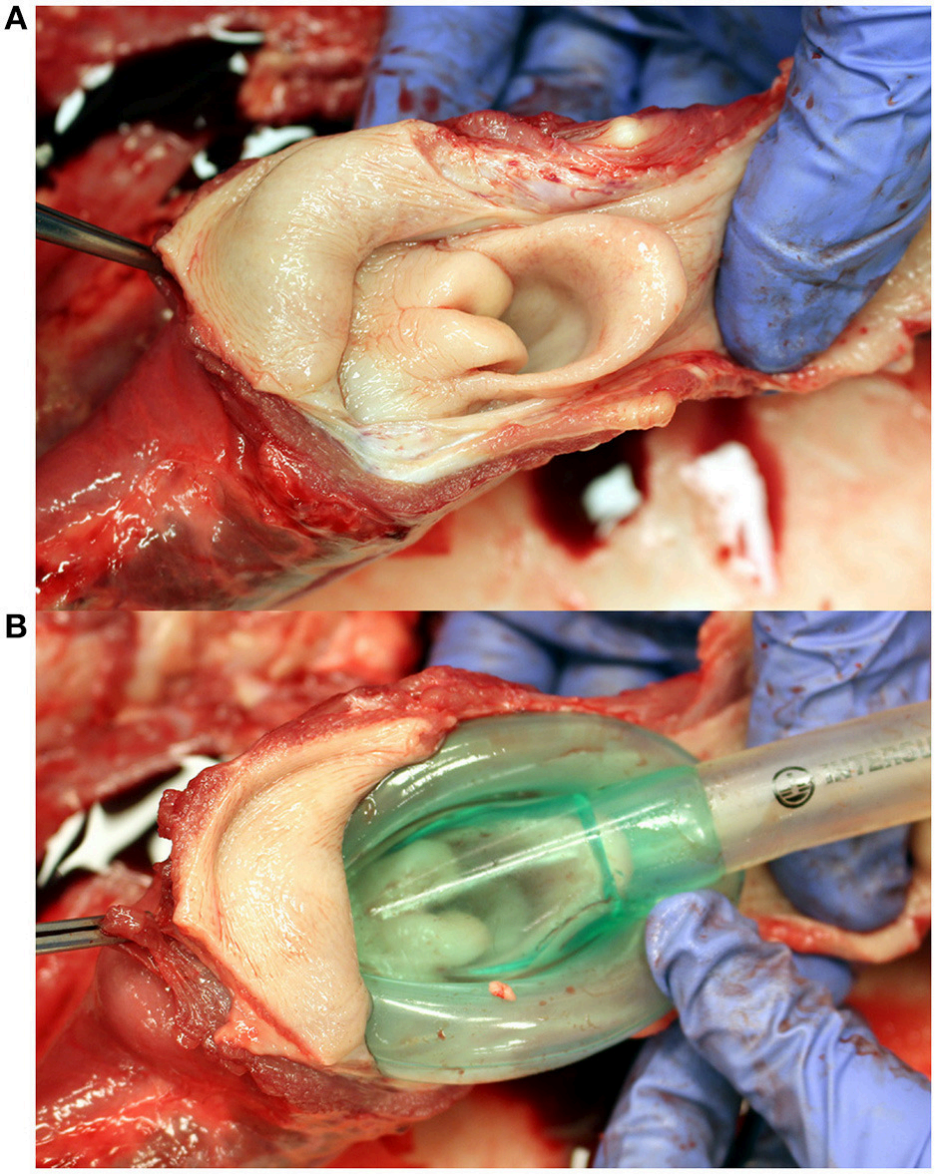
Dissection of a neonatal calf to to demonstrate placement of a laryngeal mask airway (LMA). **(A)** Laryngopharynx and laryngeal entrance; dorsal view. **(B)** Placement of the LMA over the laryngeal entrance; dorsal view. Note that the distal tip of the LMA occupies the laryngopharynx / esophageal entrance.

**Figure 3:**
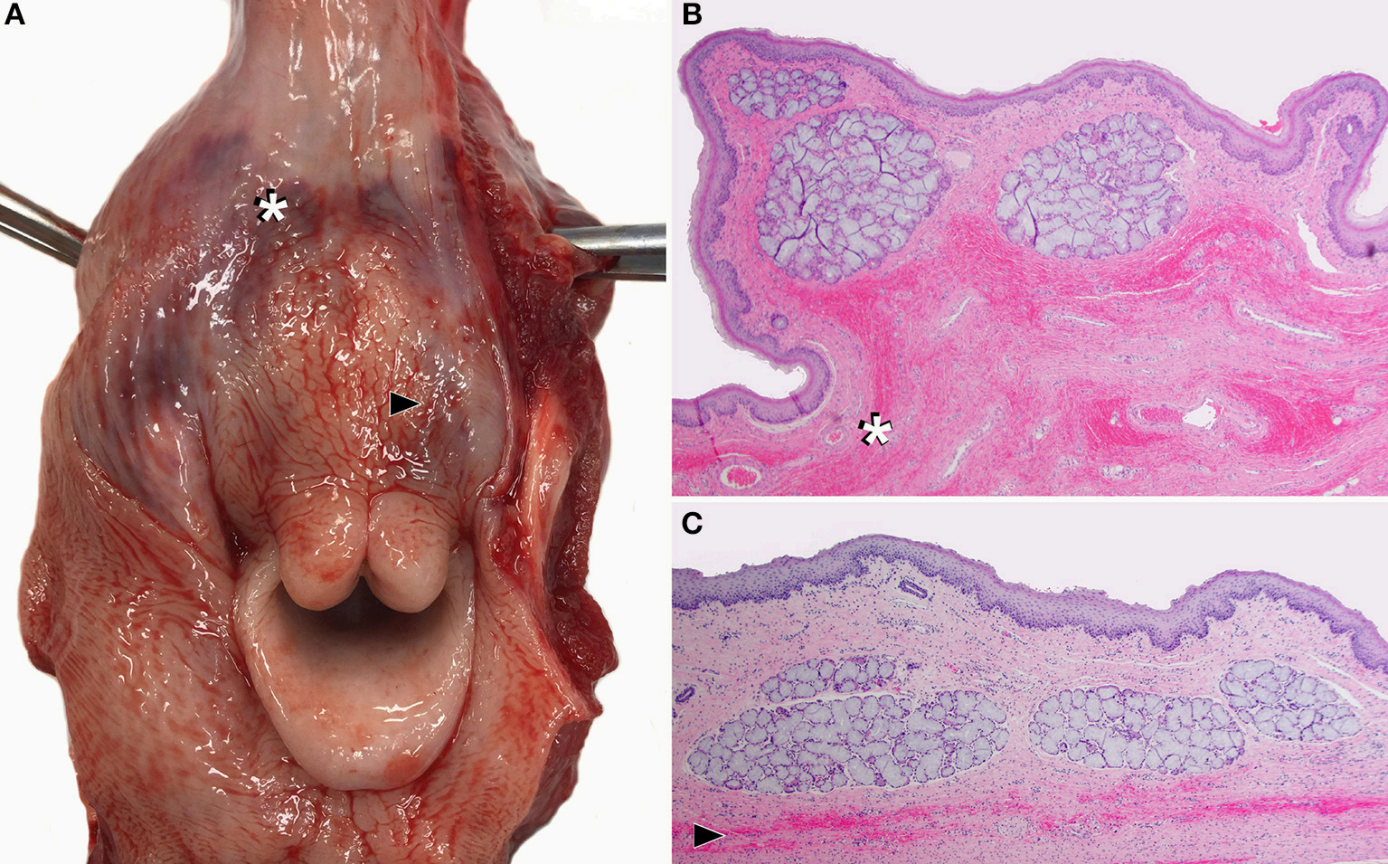
Dissected larynx of a neonatal calf (Calf 8), showing the most severe lesions caused by laryngeal mask airway (LMA) use among the nine calves in this study. **(A)** Within the laryngopharynx, there is moderate submucosal hemorrhage (bruising / hematoma) with no ulceration, where the cuff of the LMA would occlude the esophageal entrance. Two transverse histologic sections taken at the sites of submucosal hemorrhage indicated by: **(B)** the white asterisk and **(C)** black arrow head in **(A)**. Hemorrhage appears as linear bands or patches of free red blood cells (white asterisk and black arrowhead) dispersed through submucosal tissue. The overlying epithelium is intact and uninjured.

Placement of the selected LMA is shown in Figure 2. On post mortem examination, no or minimal evidence of trauma was visible in the oropharynx or laryngopharynx of Calves 1, 2, 3, 5, 6 and 7. Calves 4, 8 (Figure 3), and 9 had mild to moderate submucosal hemorrhage within the laryngopharynx, where the cuff of the LMA would occlude the esophageal entrance. Histologically, there was no evidence of mucosal ulceration in any section. In sections in which submucosal hemorrhage was present, it was deemed inconsequential and unlikely to have caused the calves any undue discomfort had they continued to live.

## Discussion

This proof-of-concept study shows that the Solus® LMA, a device developed for use in humans, was easy to place in young calves and enabled effective ventilation. Both the LMA, and the industry standard of care, the MMR, improved PaO_2_, PaCO_2_, and SaO_2_ compared to the mean baseline values. This implies that both were successful in inflating neonatal calf lungs and correcting hypoxia and hypercapnia. Subjectively, fewer complications were encountered during LMA ventilation than during ventilation with MMR, although this was not directly quantified.

Opening airways is critical for establishing the oxygen tension necessary for initiating the circulatory changes needed for extrauterine life. Calves that undergo a dystocia are born weak and are often unable to self-ventilate, resulting in respiratory acidosis, hypoxemia, hypothermia, and perinatal mortality (3). It is imperative that positive pressure ventilation is initiated to overcome the surface tension of unexpanded lungs (11, 18). The LMA has been successfully used to ventilate the lungs of humans, cats, dogs, pigs, and rabbits (12, 14–17). Similarly, the LMA successfully ventilated 2- to 11- day old calves in the present proof-of-concept study. Although further field studies are required, it is expected that the LMA could be a valuable tool for newborn calf resuscitation due to its ability to successfully inflate neonatal calf lungs.

Previously, a human continuous positive airway pressure machine with facial mask was assessed for use in newborn calves with pharmacologically-induced respiratory insufficiency, in a similar study design to the one presented here. However, it only provided modest benefit and required specialized equipment (18). The LMA and BVM system used in the present study effectively ventilated the calves and the equipment is easily acquired, compact, and portable, and requires minimal maintenance. Unfortunately, both methods require two people, which may limit their usefulness in some situations such as on farm when only one person is available.

This study used nine neonatal Holstein bull calves. The calves were all <11 days of age, but they were not newborn and did not have a reported history of dystocia. This proof-of-concept study was designed to determine if an LMA had adequate anatomic fit and could be used to ventilate neonatal calves. Now that this has been established, further work is needed using newborn calves that are apneic and acidemic from dystocia. Slight differences in body size might impact the anatomic fit of the LMA so the size 3 Solus® device might need to be considered for younger, smaller calves.

A limitation of this study was the randomization of the order of ventilation with the LMA and MMR. Data was collected for the calf that regurgitated (Calf 6), but aspiration was confirmed during necropsy and thus, the calf was removed from the dataset. Similarly, when ventilated with the MMR, several of the other calves had become subjectively more bloated than when they were ventilated with the LMA, suggesting a degree of aerophagia occurred with the MMR, although this was not explicitly examined during Stage Three. This may indicate an advantage of the LMA over the MMR, in that fewer complications were encountered when calves were being ventilated with the LMA. This should be interpreted with caution, however, based on the small numbers of calves and observational nature of this conclusion. The LMA device is designed to protect the airway whereas the MMR is not.

Another limitation was the inability to collect accurate data on tidal volume. Because of severe air leakage around the muzzle of the calves when using the MMR, this would not have provided accurate or meaningful information. Although mechanical ventilation could have controlled the variability in ventilation, a BVM was selected due to its relevance for field use. However, it is important to note that because tidal volume was not measured nor controlled, this may have impacted the measured blood parameters.

This proof-of-concept study demonstrated for the first time that an LMA can successfully be used to ventilate anesthetized neonatal calves. The LMA shows promise for ventilating apneic, newborn calves in a field setting, as the LMA was simple to place and was able to effectively aerate calf lung tissue. However, field studies using clinical dystocia cases are required.

## Ethics statement

This study was carried out in accordance with the recommendations of Canadian Council on Animal Care. The protocol was approved by the University of Calgary's Veterinary Science Animal Care Committee.

## Author contributions

CW was the principle investigator for this project. LA wrote the project proposal with guidance from CW, NC, and SB. All authors participated in study design, data collection and analysis, interpretation of the results, and preparation of the manuscript.

### Conflict of interest statement

Larygneal mark airway devices were provided in-kind from both Intersurgical Ltd. and Teleflex Medical Europe Ltd. Neither company was involved in study design, data collection or analysis, or preparation of the manuscript. The authors declare that the research was conducted in the absence of any commercial or financial relationships that could be construed as a potential conflict of interest. The reviewers KI and HG and the handling Editor declared their shared affiliation.
